# Translocation Biosensors – Cellular System Integrators to Dissect CRM1-Dependent Nuclear Export by Chemicogenomics

**DOI:** 10.3390/s90705423

**Published:** 2009-07-09

**Authors:** Verena Fetz, Shirley K. Knauer, Carolin Bier, Jens Peter von Kries, Roland H. Stauber

**Affiliations:** 1 Department of Molecular and Cellular Oncology, University of Mainz, Langenbeckstrasse 1, 55101 Mainz, Germany; E-Mail: fetzv@uni-mainz.de (V.F.); bier@uni-mainz.de (C.B.); 2 Leibniz-Institut für Molekulare Pharmakologie, Berlin, Robert-Rössle-Str. 10, 13125 Berlin, Germany; E-Mail: kries@fmp-berlin.de (J.P.V.-K.)

**Keywords:** chemical biology, cancer, Exportin 1/CRM1, HIV-1 Rev, import, LMB, nucleocytoplasmic transport, nucleoporin

## Abstract

Fluorescent protein biosensors are powerful cellular systems biology tools for dissecting the complexity of cellular processes with high spatial and temporal resolution. As regulated nucleo-cytoplasmic transport is crucial for the modulation of numerous (patho)physiological cellular responses, a detailed understanding of its molecular mechanism would open up novel options for a rational manipulation of the cell. In contrast to genetic approaches, we here established and employed high-content cellular translocation biosensors applicable for dissecting nuclear export by chemicogenomics. A431 cell lines, stably expressing a translocation biosensor composed of glutathione S-transferase, GFP and a rational combination of nuclear import and export signals, were engineered by antibiotic selection and flow cytometry sorting. Using an optimized nuclear translocation algorithm, the translocation response could be robustly quantified on the Cellomics Arrayscan^®^ VTI platform. Subsequent to assay optimization, the assay was developed into a higher density 384-well format high-content assay and employed for the screening of the 17K *ChemBioNet* compound collection. This library was selected on the basis of a genetic algorithm used to identify maximum common chemical substructures in a database of annotated bioactive molecules and hence, is well-placed in the chemical space covered by bioactive compounds. Automated multiparameter data analysis combined with visual inspection allowed us to identify and to rationally discriminate true export inhibitors from false positives, which included fluorescent compounds or cytotoxic substances that dramatically affected the cellular morphology. A total of 120 potential hit compounds were selected for Cellomics Arrayscan^®^ VTI based rescreening. The export inhibitory activity of 20 compounds effective at concentrations < 25 μM were confirmed by fluorescence microscopy in several cell lines. Interestingly, kinetic analysis allowed the identification of inhibitors capable to interfere with the export receptor CRM1-mediated nuclear export not only in an irreversible, but also in a reversible fashion. In sum, exploitation of biosensor based screening allows the identification of chemicogenomic tools applicable for dissecting nucleo-cytoplasmic transport in living cells.

## 1. Introduction

### 1.1. Cellular Biosensors

According to the International Union of Pure and Applied Chemistry (IUPAC; http://www.iupac.org), a biosensor is a device that uses biochemical reactions mediated by isolated enzymes, organelles or whole cells to detect the effects of chemical compounds by electrical, thermal or optical signals. However, for many biological questions sensors that detect only one parameter *in vitro* appear to be inefficient for dissecting the regulation of complex biological systems. The complexity of living organisms causes the need for a new class of sensors capable of integrating and interpreting multiple parameters into simple read outs. Such a need is also reflected by the recent shift in focus from the single gene, single target, single pathway and single drug paradigm to a more “systems biology” perspective, not only in basic science but also in drug development (details in [1,2]).

As cellular biosensors have the advantage of acting in a physiological and/or pathophysiological environment (e.g., in cancer cells), these are beginning to be widely used in cell and molecular biology to define the dynamics of cellular regulation in time and space, especially when combined with automated multi-parameter imaging technologies ([1,3,4] and references within). Also, the dramatic increase in the use of cell-based assays during all major steps of drug discovery and development has increased the demand for novel cellular biosensors. Such biosensors are expected to allow the detection of a wide variety of signaling molecules in real-time and hence, are bearing the potential for novel assay applications. Intensifying the use of kinetic, compared with snapshot, cell screening assays is expected to reveal subtle, but discrete effects of compounds, aiding the interpretation of their mode of action and leading to an improved understanding of key regulatory cellular pathways.

During the last years cell based high content screening (HCS) has evolved so that the throughput is high enough even for current primary high throughput screening (HTS) applications, measuring the temporal and spatial responses of cells to drugs and biological treatments (see [5]). The information gained from these high-content assays can be used to build a knowledge base from which better decisions about potential new lead compounds can be made early in the drug discovery pipeline based on “functional cellular responses” [1].

The ability to automate the capture and analysis of fluorescent images of thousands of cells in the wells of microtiter plates has made fluorescence microscopy one of the premier tools of cell biology, compatible with drug discovery [6]. Individual and population average measurements can be made rapidly on adherent cells with whole plate readers enabling the rapid measurement of a variety of treatments as well as fast kinetic measurements of treatment. Whereas both luminescent and fluorescent reagents have been successfully applied to a variety of functional measurements, fluorescence-based reagents have dominated so far due to the specificity, sensitivity, and temporal detection possible in sub-second time domains. However, the combined use of luminescent and fluorescent sensors will certainly be optimal to accelerate new discoveries and enable improved high content screening in the future (see [5]).

Often, redistribution approaches, a cell-based assay technology that uses protein translocation as the primary readout have been used to study the activity of cellular signaling pathways and other intracellular events. Protein targets are labeled with autofluorescent proteins (e.g., the green fluorescent protein – GFP), and stably expressing cell lines are generated. The assays are read using a high-throughput, optical microscope-based instrument, several of which have become available commercially. Such assays can be formatted as agonist assays, in which compounds are tested for their ability to promote protein translocation, or as antagonist assays, in which compounds are tested for their ability to inhibit protein translocation caused by a known agonist. Protein translocation assays have the potential for high-content, high-throughput assays for the profiling of lead series, primary screening of compound libraries, or even as readouts for gene-silencing studies [7,8]. However, any realistic applications of high-content and high-throughput cell based assays critically depend on robust and reliable biological readout systems with a high signal to noise ratio. In this context, the spatial and functional division into the nucleus and the cytoplasm marks not only two dynamic intracellular compartments vital for the cell but that can also be easily distinguished by microscopy.

### 1.2. Nucleo-Cytoplasmic Transport

**Figure 1.**
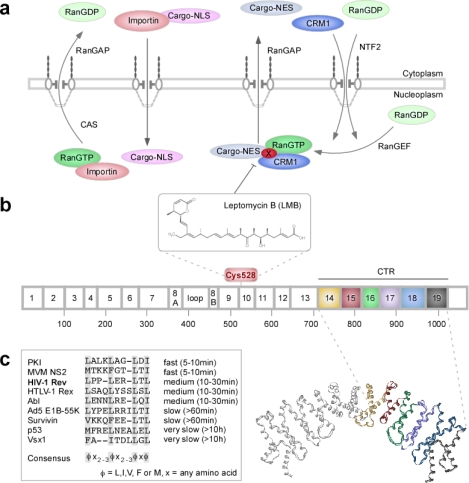
(a) Schematic illustration of the regulation of nucleo-cytoplasmic transport. As an example for nuclear export, the CRM1-dependent pathway is depicted. (b) Heat-repeat map of CRM1, which is predicted to contain 19 repeats separated by a loop within repeat 8. (c) Kinetic classification of NES sequences established by microinjection experiments of recombinant GST-NES-GFP substrates into the nucleus. Amino acids essential for function are highlighted in grey, putative consensus sequence for leucine-rich NES is shown below. (a) NLS-bearing cargoes are imported via binding to importins. In the nucleus, the importin-cargo complex dissociates upon Ran_GTP_ binding. CAS exports the respective importin back into the cytoplasm. RanGAP hydrolyses Ran_GTP_ to Ran_GDP_ resulting in release of the importin from CAS. Leucine-rich NES-bearing cargoes bind to CRM1 in the presence of Ran_GTP_, and the complex translocates into the cytoplasm, where the cargo is released following hydrolysis of Ran_GTP_ to Ran_GDP_. Ran_GDP_ is reimported by NTF2 and reconverted into Ran_GTP_ by RanGEF. Leptomycin B (LMB) inactivates CRM1 by covalent binding to Cys_528_, thereby irreversibly inhibiting NES-mediated nuclear export. For the selective export of cargoes, NES-specific cofactors (X) have been suggested. RanGEF - Ran guanine nucleotide exchange factor, RanGAP - Ran GTPase activating protein 1, NTF2 - Nuclear Transport Factor 2, CAS - cellular apoptosis susceptibility, X – putative NES-specific cofactor. (b) Heat repeat 10 contains the cysteine residue 528, which is covalently modified by LMB. The C-terminal region (CTR) comprises heat repeats 14 to 19, colored from yellow to black. The CTRs of two proteolytic fragments of CRM1 (residues 707–1027, PDB 1W9C) are displayed as a dimeric solid ribbon representation of the backbone superposition (heat repeats of one fragment colored as above).

The eukaryotic cell is divided into distinct reaction compartments, which enables the fine-tuning of biochemical processes being fundamental for cellular specialization and thus, for the development of higher eukaryotes. However, the full exploitation of this regulatory potential necessitates the controlled intracellular localization and transport of proteins and macromolecules (see [9]). In particular, the integration of signals from the (extra)cellular microenvironment into adaptive cellular responses, including the modulation of genetic programs, critically depends on regulated nucleo-cytoplasmic transport (Figure 1a) [10,11]. The nucleus is separated from the cytoplasm by the nuclear envelope bilayer, which can only be passaged through the nuclear pore complexes (NPCs), large macromolecular machines of ∼125 MDa (details in [12–14]). The NPC can be divided into cytoplasmic fibrils, a central core and the nuclear basket. Major structural and functional constituents of the NPC are the so called nucleoporins, a group of proteins with ∼30 known members (references in [12,13]).

The NPC is a selective barrier for molecules larger than 40–60 kDa and allows transport of cargoes with diameters up to 40 nm [14,15]. One NPC can perform up to 1,000 translocation events per second, which can also occur against a concentration gradient, and is mediated by soluble transport factors that in turn shuttle between the nucleus and the cytoplasm ([14] and references within). Importantly, even molecules that are theoretically small enough for passive diffusion are actively and selectively transported (see [3,14]). Regulated transport of most proteins and several RNA species [10] is mediated by karyopherins, belonging to the superfamily of importin β-related proteins [16]. Karyopherins are also named importins and exportins according to their catalyzed mode of transport. It is striking that the 20 karyopherin members are capable of mediating the controlled transport of the huge variety of cargoes, which differ dramatically with respect to their biochemical and -physical properties. Hence, the exact mechanism ensuring the flexibility and correct regulation of nuclear transport are still not resolved, but appear to rely on a complex network of dynamic protein interactions involved in the formation of general and selective transport complexes as well as in the passage through the NPCs itself (see [11,17].

In contrast to receptors, which interact with only one or a few structurally related cargoes, import receptors such as importin α/β or the export receptor CRM1 (chromosome region maintenance)/exportin1 (Figure 1b), transport numerous proteins and/or ribonucleoprotein complexes by recognizing specific signal sequences present in the cargo and/or associated adapter proteins (see [14,18]). Importins bind to short stretches of basic amino acids, termed nuclear localization signals (NLS). The best characterized nuclear export signals (NESs) are hydrophobic and consist of a short leucine-rich stretch of amino acids (Figure 1a/c) [3,14,18]. Although also CRM1-independent pathways are crucial for cellular homeostasis, CRM1-independent NES are less well characterized (for details see [3,14,17,18] and references within). Recognition of the NES by the CRM1 receptor is specifically inhibited by LMB, which binds covalently to Cys_528_ of CRM1 (Figure 1b) [19]. The driving force of active nuclear transport appears to be the Ran_GTP_ (Ran = Ras-related nuclear protein) gradient between the nucleo- and the cytoplasm. CRM1 cooperatively binds Ran_GTP_ and the NES-containing cargo in the nucleus. Subsequent to transport through the NPC, Ran_GTP_ is hydrolyzed in the cytoplasm into Ran_GDP_ by the Ran GTPase-Activating Protein 1 (RanGAP), thereby triggering conformational changes resulting in cargo release. Ran_GDP_ is reimported into the nucleus by the Nuclear Transport Factor 2 (NTF2) and converted into Ran_GTP_ by the nuclear Ran Guanine Nucleotide Exchange Factor (RanGEF/RCC1 - regulator of chromosome condensation 1). Importins depend on Ran_GTP_ to release their cargo in the nucleus and to be retransported into the cytoplasm by the export receptor Cellular Apoptosis Susceptibility (CAS) (for details see [14,18]).

Nuclear transport in general is regulated by versatile signals such as hormones, cytokines, growth factors, cell cycle signals, developmental signals or stress. Hence, regulated nucleo-cytoplasmic transport is accepted as an efficient way to control the activity of a variety of proteins involved in physiological and pathophysiological processes [20,21]. As such, NLSs as well as leucine-rich NESs have been identified in an increasing number of disease relevant cellular and viral proteins implicated in transcription control, cell cycle control, mitosis and RNA transport (see [10,22–24]).

Regulation can either occur through the expression status of transport components and/or at the level of the transported cargo itself. Dynamic exposure of transport signals due to conformational changes triggered by transient post-translational protein modifications, such as phosphorylation or acetylation, are known to modulate transport and the biological activities of proteins ([25] and references within). Interestingly, even isolated NESs can be grouped into specific kinetic classes according to their activity *in vivo* (Figure 1c), which appear to correlate with the biological activity of the proteins the NESs are originating from [23,26].

As regulated subcellular localization provides also an attractive way to rationally control the activity and stability of regulatory proteins and RNAs, targeting nucleo-cytoplasmic transport as a novel therapeutic principle has attracted major interest by academia and industry (see [20,23]). However, despite intense investigation the detailed molecular mechanism regulating the complex orchestration of nucleo-cytoplasmic transport is still not understood. Thus, in addition to genetic depletion strategies and sophisticated *in vitro* models employing the recent advances of nanotechnology [12,15], novel approaches such as chemogenomic screens will most likely aid in dissecting regulatory mechanism and contribute to clarifying cargo specificity.

### 1.3. Chemogenomics

Chemogenomics is the study of the interaction of biological systems with exogenous small molecules, i.e., analyzing the intersection of biological and chemical spaces (see [27,28]). Biological entities can be isolated proteins or more complex systems, such as living cells, which can be analyzed at the genomic, transcriptomic, proteomic or phenotypic level. The chemical space of small molecules consists of the current commercial and academic collections of compounds, and the virtual space, represented by all compounds possibly synthesizable. A major challenge of cheminformatics is therefore to charter the virtual space of small molecules using realistic biological constraints (bioavailability, druggability, structural biological information). Small molecules that allow a chemical knock out of a cellular reaction or a cell phenotype can be selected by phenotypic screens, and used as molecular tools to identify previously uncharacterized proteins and/or molecular mechanisms responsible for the given phenotype. This experimental approach termed forward chemogenomics (“from small molecules to targets”) defines chemogenomics at the intersection of the biological space with that of “small molecules”, rather than with the space of “drugs” that must comply with the restrictive approval conditions for therapeutics [27]. Consequently, we here describe and employ translocation biosensors as cellular system integrators to dissect nuclear export by chemogenomics.

## 2. Results and Discussion

### 2.1. The RevNES-Biosensor

**Figure 2.**
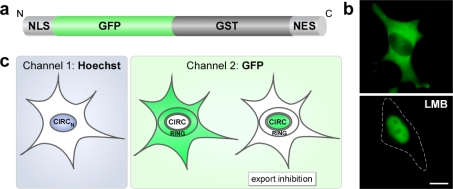
(a) Schematic representation of the modular RevNES-biosensor. (b) In living HeLa cells transiently expressing the RevNES-biosensor, the protein localized predominantly to the cytoplasm, whereas treatment with LMB resulted in its nuclear accumulation. Scale bar, 10 μm. (c) Schematic illustration of the masks used to mark the nucleus and the cytoplasm in order to define the cytoplasmic to nuclear translocation of the autofluorescent biosensor (details see text).

The applications of translocation biosensors for high-throughput cell based assays critically depend on robust and reliable biological readout systems with a high signal to noise ratio. Previously, we showed that the intracellular localization of a GST-GFP fusion strictly depends on the presence of transport signals and is not disrupted by passive diffusion [29]. In addition, GST-GFP is highly fluorescent, non-toxic and stable. To design a GFP-GST shuttle protein with a predominantly cytoplasmic steady-state localization, an appropriate combination of NLS and NES had to be used. Based on our kinetic classification studies (Figure 1c) [26], we combined the SV40 large T-antigen NLS (NLS) and the NES from the HIV-1 Rev protein (RevNES), to generate plasmids allowing the ectopic expression of a NLS-GFP/GST-RevNES fusion protein (RevNES-biosensor) (Figure 2a). Transient expression in several cancer cell lines confirmed that the RevNES-biosensor localized predominantly in the cytoplasm, was efficiently shuttling between the nucleus and the cytoplasm, and accumulated in the nucleus upon treatment with the CRM1 inhibitor LMB (Figure 2 b/c, and data not shown).

Hence, the system appeared to be applicable for high-throughput redistribution screening applications. For this purpose, the nucleus of interphase cells was defined as the “CIRC_N_” region by staining with the nucleic acid dye Hoechst 33342 (channel 1). In the GFP-channel (channel 2), the CIRC_N_ mask was eroded to reduce cytoplasmic contamination within the nuclear area, and the reduced mask (CIRC) was used to quantify the amount of GFP fluorescence before and after export inhibition within the nucleus (Figure 2c). Subsequently, the nuclear mask was dilated to cover as much of the cytoplasmic region as possible without going outside the cell boundary. Removal of the CIRC region from this dilated mask creates the RING mask covering the cytoplasmic region outside the nuclear envelope (Figure 2c).

### 2.2. Generation and Characterization of Cell Lines Stably Expressing the RevNES-Biosensor

**Figure 3.**
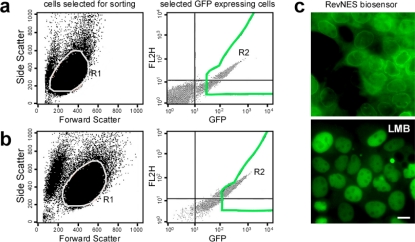
Generation and characterization of RevNES-biosensor expressing cell lines. (a/b) A431 were transduced with pINCO_Rev-sensor containing retroviruses and subjected to two rounds of FACS sorting. A distinct cell population based on cell size (gate R1, left panels) and GFP fluorescence intensity (gate R2, right panel), was isolated. For the second round of FACS, only cells robustly expressing the RevNES biosensor were selected (b, R2 right panel). (c) Fluorescent microscopic images of the A431_bio_ cell population isolated after two rounds of FACS. All cells showed a high expression of the RevNES-biosensor, which localized predominantly in the cytoplasm with some accumulation at the nuclear membrane (left panel). Treatment with LMB (10 nM; 2h) resulted in almost complete accumulation of the biosensor in living cells. Scale bar, 10 μm.

For high-throughput cellular assays it is time consuming and costly to continuously generate cells transiently expressing the biosensor by transfection. Thus, we engineered cells stably expressing the RevNES-biosensor by retroviral transduction of the epithelial carcinoma cell line A431. Subsequently, a highly fluorescent cell population was established by G418-selection and enriched by two rounds of fluorescence activated cell sorting (FACS, Figure 3a/b). The stable expression as well as the efficient cytoplasmic to nuclear redistribution of the biosensor was controlled in the resulting A431_bio_ cell line by fluorescence microscopy prior and subsequent to LMB treatment (Figure 3c). Upon cultivation in selection media, expression of the biosensor was stable for at least four months (data not shown).

### 2.3. Assay Development on the Cellomics ArrayScan® VTI Imaging Platform

**Figure 4.**
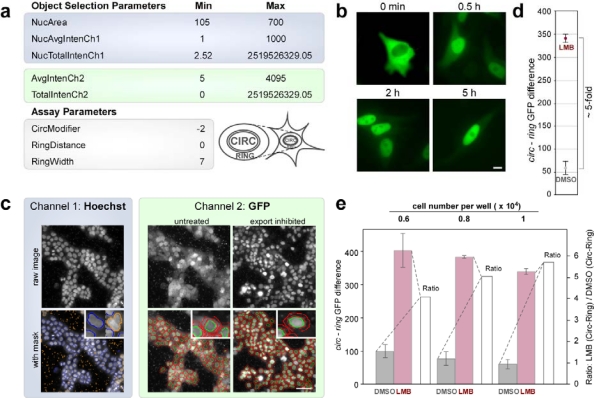
(a) Optimized object selection parameters (min/max settings) for channel 1 and 2 (highlighted in blue and green, respectively). (b) LMB treatment (10 nM) of A431_bio_ cells resulted in a rapid nuclear accumulation of the biosensor already after 30 min, which remained stable also after 5 h without obvious cytotoxic effect on cell morphology. Scale bar, 10 μm. (c) Translocation assay analysis of A431_bio_ cells using the Cellomics Arrayscan^®^ VTI platform. The Hoechst and GFP signals were recorded in channel 1 and 2 (highlighted in blue and green), respectively, following LMB treatment (10 nM, 5 h). In the upper panel, the original raw images are shown. The lower panel shows the overlay with the respective masks. In channel 1, cells selected for further analysis are outlined in blue, cells rejected due to their size or shape are outlined in orange. In channel 2, the CIRC mask and RING region are outlined in green or red, respectively. Scale bar, 50 μm. (d) Average signal difference (CIRC-RING) of LMB treated cells (10 nM, 5 h) versus DMSO assay controls in one representative 384-well plate. 1 × 10^4^ cells/well were seeded, and the values were derived from analyzing ∼500 cells/well (see Table 1 for details) revealing a translocation index of > 5-fold. (e) Differences in assay performance depending on cell density. The indicated numbers of A431_bio_ cells were seeded into 384-well plates. Sixteen h later, cells were treated with LMB (10 nM) or DMSO as a control for 5 h, and the translocation index quantitated using the Cellomics Arrayscan^®^ VTI imager. CIRC-RING differences were plotted for DMSO control (grey) and LMB treated (red) cells (left scale). The ratio of these values (white columns) performed best for 1 × 10^4^ seeded cells/well. Columns, mean; bars, SD.

To perform HTS the assay had to be adapted to the 384-well plate format. For this purpose, the molecular translocation assay was adjusted by modifying several parameters to ensure an optimal object identification (Figure 4a). The first step was to adjust the background correction and to define the threshold of pixels derived from the Hoechst 33342 signal. This calculation resulted in an optimized object identification, capable to automatically excluding “non cellular” irregular objects (too small/big, debris, compound aggregates, etc.) in channel 1. By adjusting object segmentation parameters, the fitting of the nuclear mask to the Hoechst 33342 signal was further optimized. Also for channel 2, the background correction and values for the threshold of the GFP signal were defined to exclude irregular and potentially false positive signals from the analysis. The translocation index is calculated as the difference of the nuclear intensity minus the cytoplasmic ring intensity (CIRC-RING) on a single cell basis. Of note, these parameters might have to be further adapted to the morphology of the cell type used for screening.

In order to select an appropriate time point for assay execution, we next examined the kinetics of export inhibition caused by LMB, a drug that efficiently prevents the CRM1/NES-interaction by covalently binding to Cys_528_ of CRM1 (Figure 1b) [19]. As shown in Figure 4b, LMB treatment of A431_bio_ cells caused rapid export inhibition resulting in almost complete nuclear accumulation of the biosensor already after 30min. A similar kinetic of inhibition was observed when cells were seeded in 384-well plates and analyzed using the Cellomics ArrayScan^®^ VTI imager (Figure 4c). Addition of LMB resulted in a ∼five-fold increase of the translocation index (CIRC-RING) in a roughly linear fashion over time, which remained stable after two to at least five hours post treatment (Figure 4d, and not shown). LMB is known as a “fast acting” irreversible export inhibitor [30], which we found not to cause obvious cytotoxic effect even after a 5 h treatment period (Figure 4b). Of note, prolonged incubation with LMB was toxic for the cells as demonstrated by analyzing cell viability (Suppl. Figure 1a). Since our goal was to also identify export antagonists with slower and thus, potentially different biochemical modes of action, 5 h was selected as the standard incubation time for the screening of the compound library.

To finally establish the optimal assay cell density, A431_bio_ cells were seeded in 384-well plates at 0.6, 0.8 and 1 × 10^4^ cells per well. Cells were treated with 10nM LMB or the DMSO control (0.4% final concentration), and analyzed using the Cellomics ArrayScan^®^ VTI imager to quantify the translocation response at all three seeding densities (Figure 4e). Employing our optimized object selection and assay parameters (Figure 4a), the cell density established by seeding 1 × 10^4^ cells resulted in an optimal translocation index and thus, was selected for screening. Of note, the low DMSO concentrations used (0.4% final concentration) did not induce any detectable cellular effects when compared to PBS (not shown).

### 2.4. Biosensor-Based HTS for Export Inhibitors

Subsequent to the evaluation and optimization of the assay parameters, the A431_bio_ cells were used to screen the 17K *ChemBioNet* compound collection in a 384-well plate format employing the Cellomics ArrayScan^®^ VTI platform. The *ChemBioNet* compound collection used for screening was selected according to the Lipinski rules (“Rule of five”) [31]. In the context of the *ChemBioNet* library, these rules were used to select molecules predicted of being able to pass through biological membranes, including the plasma as well as the nuclear membrane and thus, are suitable for cellular assays. Based on the assay signal window and Z’-factor data (Table 1), the active criterion for the primary screen was set at a compound translocation index > 2 (T_i_ = compound_(CIRC-RING)_/DMSO_(CIRC-RING)_). Each plate contained a row with DMSO treated control cells and a row with LMB treated cells allowing to define the dynamic assay range for the individual plates and to also monitor the robustness of the assay. Only valid objects, i.e., cells that pass the object selection criteria (Figure 3a) were included in the analysis.

In the course of the screen, a total of 16,671 compounds were tested at a final concentration of 25 μM (corresponding to a DMSO concentration of 0.4%) for their ability to inhibit export of the RevNES-biosensor. The complete HTS assay was performed in five days. The total scan time for the primary screen on the Cellomics ArrayScan^®^ VTI platform was 56 h, the mean scan time/well was 11 s, and the average number of valid objects (= cells) was 470 (Table 1). Although we used stringent parameters to optimize automated object identification and exclusion of “non cell objects” together with our translocation algorithm, the primary assay identified ∼500 events as “potential hits”, causing a T_i_ > 2.

**Figure 5.**
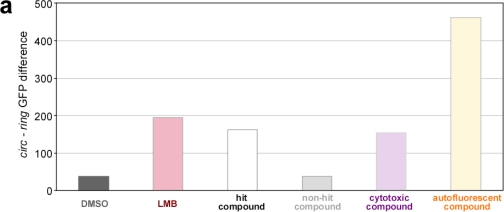

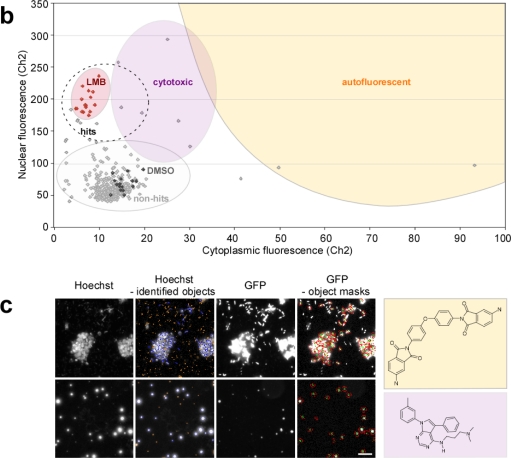
Intersection of the biological and chemical space. Translocation index-based analysis for cytotoxicity and fluorescence interference. (a) CIRC-RING values representative for negative and positive control substances (DMSO, LMB), export antagonists, substances showing no effect on export, cytotoxic and autofluorescent compounds obtained from the analysis of a representative 384-well plate. (b) Scatter plot of the nuclear intensity/well versus the cytoplasmic intensity/well in the “green” target channel 2 (GFP) subsequent to compound exposure to mark the intersection of the chemical and biological space. Values from a representative 384-well plate are shown. The DMSO controls (dark grey) are grouped together with the non-hit compounds (light grey), and are well separated from the LMB controls (red) and a potential hit population (black dotted circle). Potential hit compounds partially overlap with cytotoxic substances (violet), identified by visual inspection. Autofluorescent compounds (orange) usually display high fluorescence intensities and are well separated. (c) Representative images from compound wells that exhibited either a dose-dependent autofluorescence (upper panel) or a dose-dependent cytotoxicity (lower panel). Raw images and mask overlays are shown for both channels. In the Hoechst channel, objects selected for further analysis are outlined in blue, objects rejected are outlined in orange. In the “GFP channel”, the green outline represents the nuclear region mask, and the red outline the cytoplasmic ring region. Scale bar, 50 μm. The chemical structure of the respective compounds are shown on the right.

**Figure 6.**
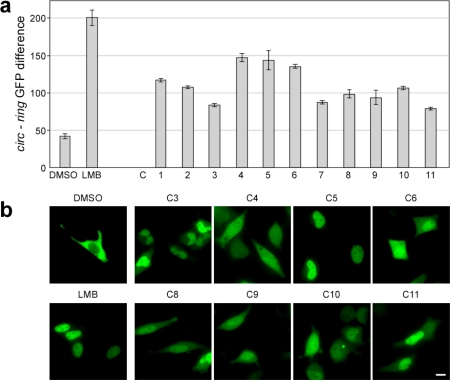

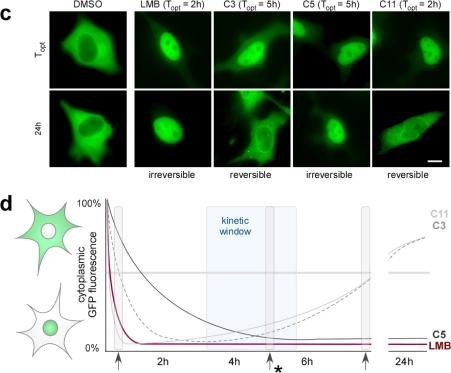
(a) Secondary ArrayScan^®^-based validation of potential export antagonists. 1 × 10^4^ A431_bio_ cells/well were seeded into 384-well plates. 16 h later, cells were treated with LMB (10nM), DMSO or the indicated compounds (C1-C11; 25 μM) for 5 h, and translocation was quantitated using the Cellomics ArrayScan^®^ VTI imager. The CIRC-RING differences are indicative of efficient export inhibition. Columns, mean; bars, SD. (b) In lab verification of hit compound performance in living HeLa cells transiently expressing the RevNES biosensor. Indicated compounds were applied at a concentration of 25 μM for 5 h, resulting in efficient nuclear accumulation of the biosensor. Scale bar, 10 μm. (c) Compound specific kinetics of inhibition. HeLa cells transiently expressing the RevNES biosensor were treated with LMB (10nM) or the indicated compounds (25 μM). The localization of the biosensor is shown at the indicated time points when the compounds displayed their maximum inhibitory activity (top panel), as well as 24 h post treatment. The inhibitory activity of C3 and C11 was reversed after 24 h. Scale bar, 10 μm. (d) Kinetic activity profile of export inhibitors. Selection of different time points (indicated by the arrows) for snapshot analysis (highlighted in grey) may significantly influence the number and type of compounds identified in primary screens. A continuous kinetic monitoring of the biosensor translocation may ensure the identification of the maximum number of bioactive substances and the optimal extraction of informations concerning their bioactivity. Time point used for analysis of the primary RevNES-biosensor screen is marked by the asterisks. An optimal kinetic window is highlighted in blue.

To separate “true” from “false positive” hits, we made use of the ArrayScan^®^ view software. This option to reinspect the actual images from the plate controls, empty wells or the compound wells is a major advantage of the Cellomics ArrayScan^®^ VTI microscopy-based cellular screening assay, and a clear advantage compared to enzymatic *in vitro* assays [3]. Employing the ArrayScan^®^ view software, we found that among the “potential hits” were autofluorescent substances, cytotoxic compounds (for examples, see Figure 5), or substances that triggered intracellular aggregation of the biosensor (∼380). As shown in Figure 5c (upper panel), autofluorescent substances can mimic a “true” translocation event. Likewise, cytotoxic compounds can induce apoptosis of the indicator cells, leading to loss of cell morphology, condensation of the nucleus and/or biosensor-aggregation (Figure 5c, lower panel). Cytotoxicity was primarily determined by visual inspection, and verified by analyzing cell viability for selected compounds (Suppl. Figure 1a, and data not shown). Such structures were passing the intensity and size filters selected in the algorithm. Hence, for future applications the development of additional, more stringent assay parameters is desirable, which should be used especially in secondary screen to automatically subclassify potential hit compounds. However, also compounds causing off-target effects may represent interesting and also biologically relevant chemical tools for forward chemogenomics (“from small molecules to targets”). Of note, the majority of drugs currently used as anti-cancer chemotherapeutics can be classified as “cytotoxic compounds”. Hence, it will be of general interest to dissect the molecular mechanisms by which these novel cytotoxic substances identified in our biosensor assay execute their cell damaging effects by employing *in silico* modeling as well as *in vitro* studies. From the ∼500 “potential hits” identified by the primary screen, visual inspection resulted in the selection of 120 compounds (0.7%), which were neither autofluorescent nor cytotoxic at the tested concentration (25 μM) and induced efficient nuclear export inhibition (T_i_ > 2) in a secondary Cellomics Arrayscan^®^ VTI based-screen (Figure 6a). Eleven representative compounds were subsequently ordered for a detailed “in lab evaluation” by fluorescence microscopy.

### 2.5. Functional and Kinetic Profiling of Novel Export Antagonists

These eleven compounds showed inhibitory activity not only in A431_bio_ cells on the ArrayScan^®^ platform (Figure 6a), but also caused nuclear accumulation of the RevNES-biosensor when tested in several epithelial cancer cell lines (Suppl. Figure 1b). So far, we did not observe a cell line-specific difference in their activity. Importantly, when we analyzed the kinetics of inhibition, we found that the export antagonists could be classified into fast or slow acting compounds with an irreversible or reversible mode of inhibition (Figure 6c/d). As depicted, compound C3 blocked export as fast as LMB, but the inhibition was completely reversed after 24 h (Figure 6c/d). Such a bioactivity profile suggests that C3 inactivates CRM1 not by covalent binding to the active NES-interacting pocket as shown for LMB [19], and appears to be unstable under physiological conditions. In contrast, C5 is a slow acting compound, which irreversibly inhibits nuclear export. Such a delay in export inhibition might be explained by the reduced ability of the substance to enter the cell and/or the nucleus. However, the exact molecular mechanisms and protein targets of the identified inhibitors remain to be resolved by employing comprehensive experimental approaches.

Besides the identification of novel transport inhibitors, our approach also impressively underlines the enormous advantage of kinetic biosensors compared to snapshot assays. Our biosensor assay allows to continuously monitoring the translocation process in living cells in order to identify the maximum number of potentially bioactive substances and to simultaneously extract important information concerning the cellular bioactivity and pharmacology of the compounds. The kinetic activity profile illustrated in Figure 6d demonstrates that the discovery rate and type of molecules can be rationally influenced by the kinetic window selected for assay analysis. Within drug discovery projects, this may result in a significant enrichment for hits with a desired mode of activity, e.g., fast acting inhibitors, and thus, significantly reduces the number of compounds, which have to be validated and biologically characterized by time consuming and costly assays downstream in the drug development pipeline.

## 3. Experimental Section

### 3.1. Plasmids

To construct the retroviral expression vector, pINCO_Rev-sensor, the coding region for BFP in the PINCO vector [32] was replaced by the NLS-GFP/GST-RevNES (RevNES-biosensor) expression cassette [3] by cloning into the BamHI/NotI-restriction sites. The plasmid was verified by sequence analysis according to [3].

### 3.2. Cell Culture, Transfection, Retrovirus Production and Transduction

A431 and HeLa cell lines were maintained under conditions described [33] and transfected with plasmid DNA using Lipofectamine 2000 (Invitrogen, Karlsruhe, Germany) according to [34]. Production of retroviral stocks and transduction were carried out as previously described [33].

### 3.3. Generation of Stable Cell Lines

To generate cells stably expressing autofluorescent RevNES-biosensor protein, A431 cells were transduced with pINCO_Rev-sensor. Subsequently, cells were cultured in the presence of 500 μg/mL G418 (Sigma Aldrich, Munich, Germany). A highly fluorescent cell population (A431_bio_) was isolated by fluorescence activated cell sorting (FACS), which was repeated after two weeks cultivation of the cells in G418-containing selection media as described [33].

### 3.4. Automated High Throughput/Content Screening Platform, Assay Preparation and Execution

Automated assay analysis was performed using the Cellomics ArrayScan^®^ VTI Imaging Platform (Thermo Fisher Scientific Inc., Berkshire, UK), an automated fluorescence microscopic imaging system. Biosensor expressing A431_bio_ cells were detached with trypsin (Invitrogen, Karlsruhe, Germany) and counted in a Fuchs-Rosenthal chamber (Roth, Karlsruhe, Germany) as described [3]. Cells were seeded with an electronic multichannel pipette (Eppendorf, Hamburg, Germany) into 384-well thin bottom Greiner μclear^®^ plates (Greiner, Frickenhausen, Germany) in a final volume of 50 μL cell culture medium per well. Prior to seeding, the plates were coated with poly-l-lysine (Sigma Aldrich, Munich, Germany). The compounds of the *ChemBioNet* library were added with a liquid handling robot (Sciclone ALH-3000, Caliper Life Sciences, Rüsselsheim, Germany) at a final concentration of 25 μM. Plates were incubated at 37 °C, 5% CO_2_ and 95% humidity. On each plate, DMSO (0.4%) was added to one row as a negative control and LMB (10nM) to another row as a positive control. After 5 h of incubation with the compounds, cells were fixed by the addition of 50 μL 4% PFA (Sigma Aldrich, Munich, Germany) for 20 min. Subsequently, plates were washed three times with PBS using an ELISA washer (ELX405UV, BioTek, Bad Friedrichshall, Germany). Nuclei were stained by addition of Hoechst 33342 (Sigma Aldrich, Munich, Germany) at a final concentration of 40 μM for 10 min. After a final wash with PBS, 50 μL PBS were left in each well and the plates were sealed and stored at 4 °C until analysis.

### 3.5. Image Acquisition and Analysis

Images were acquired and analyzed on the Cellomics ArrayScan^®^ VTI Imaging Platform. Fluorescence images were captured as described [3]. Briefly, Hoechst 33342 (excitation 405 nm, emission 400–450 nm) and GFP (excitation 470 nm, emission 509 nm) fluorescence [35] were captured using sequential acquisition to give separate image files for each. Binary image masks were created of GFP and Hoechst 33342 positive staining to define regions of interest (ROI) for analysis. This was done by applying a median filter (3 × 3 pixel radius) to remove noise and to approximate the distribution of staining intensity to a median value. Automatic thresholding using the Isodata algorithm [36] was used to convert the image to a binary mask that included all fluorescence data above background. The Hoechst 33342 staining (channel 1) mask was used to define the nuclear ROI. Subsequently, the Hoechst 33342 mask was subtracted from the GFP mask (channel 2) to create a staining mask defining the cytoplasmic ROI. Scans were performed sequentially with settings to give sub-saturating fluorescence intensity, and a minimum of 100 valid objects per well was recorded.

### 3.6. The ChemBioNet Compound Collection

The *ChemBioNet* compound collection used for screening consists of a collection of 16,671 compounds, selected according to the Lipinski rules (“Rule of five”) [31]. For the *ChemBioNet* library, these rules were used to select molecules, which are capable of passing through biological membranes and thus, are suitable for cellular assays. The average molecular weight of a compound in the screening collection is 388 g/mol and 80% of all compounds have a molecular weight between 300 and 500 g/mol. There is no compound with more than five hydrogen bond donors and none of the compounds contains more than ten hydrogen bond acceptors. In addition, 90% of the compounds contain ten or fewer rotatable bonds. The screening library fulfils all criteria that are usually applied to select small molecules with a high bioavailability.

### 3.7. Drug Treatment and Fluorescent Imaging of Cells

Cells were treated with leptomycin B (LMB) (Sigma Aldrich, Munich, Germany) as described [37]. Compounds were purchased from ChemDiv (San Diego, CA, USA) and dissolved in DMSO (50 mM). Cell nuclei were marked by staining with Hoechst 33258 (Sigma Aldrich, Munich, Germany) as described [38]. Cells were observed as described [38], and twelve-bit black and white images captured using a digital AxioCam CCD camera (Carl Zeiss, Jena, Germany). Quantitation, image analysis and presentation were performed using the AxioVision^®^ software (Carl Zeiss, Jena, Germany). The total cellular GFP signal was measured by calculating the integrated pixel intensity in the imaged cell multiplied by the total area of the cell. The nuclear signal was similarly obtained by measuring the pixel intensity in the nucleus. The cytoplasmic signal was calculated by subtracting the nuclear signal from the total cellular signal. All pixel values were measured below the saturation limits, and the background signal in an area without cells was subtracted from all values. For in lab verification, at least 200 fluorescent cells from three separate images were examined to determine the average intracellular protein localization.

### 3.8. Measurement of Cell Viability

Cell viability was determined using the electric sensing zone method (CASY^®^ TT Cell Counter; Schärfe SystemGmbH, Reutlingen, Germany) and the CellTiter-Glo^®^ Luminescent Cell Viability Assay (Promega, Madison, WI) as described [39].

### 3.9. Statistical Analysis

Unless stated otherwise, the mean values and the standard deviations (SD) presented in the corresponding graphs are obtained from two independent experiments done in triplicate. For in lab verification, a minimum of 200 fluorescent cells from at least three separate images were examined to determine the average circ-ring GFP intensity difference as a measure for subcellular localization of the biosensor.

## 4. Conclusions

To date the detailed molecular mechanisms regulating the orchestration of nucleo-cytoplasmic transport are still not completely understood. Thus, in addition to biochemical studies systematic cell-based screens need to be applied to unravel novel biochemical regulations and to hopefully also clarify cargo specificity.

Here, we describe the establishment of a translocation-based cellular assay for HTS identification of general/specific export inhibitors, employing the Cellomics ArrayScan^®^ VTI imager. In order to avoid the contamination of “hit compounds” by substances, which indirectly interfere with transport, only the isolated NES sequence of the HIV-1 Rev protein was integrated as a model in the biosensor. Indirect transport inhibitors acting by the induction of intramolecular conformational changes in the NES-containing cargo, e.g., by blocking phosphorylation have been already identified in several screens (see [20]). In contrast, the hit compounds discovered by our strategy appear not to influence potential modifications of the biosensor protein. Importantly, the identified compounds displayed different bioactivity profiles, allowing their classification into fast or slow acting substances with an irreversible or reversible mode of inhibition.

**Figure 7.**
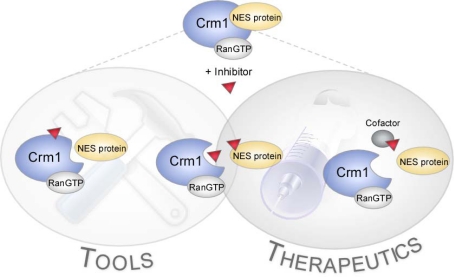
Model summarizing potential modes of action of the identified export inhibitors. Compounds may bind to CRM1 and, in an allosterical or competitive manner, affect its NES-binding domain, thereby preventing the assembly of a functional export complex. Alternatively, the compounds may directly interact with the specific NES itself or target putative cofactors required for the assembly of protein specific export complexes. Compounds displaying such a bioactivity profile would be of therapeutic interest.

Assuming that the compounds are not interacting with structural and/or functional components of the NPC (Figure 1a), several modes of action can be envisaged (Figure 7). First, similar to known export inhibitors such as LMB or N-azolylacrylates [40], the compounds may reversibly or irreversibly occupy the NES-binding pocket in CRM1, e.g., by covalently binding to critical amino acids residues, such as Cys_528_ [19]. Hence, CRM1 will be unable to accept the NES-containing cargoes for the assembly of a functional export complex with Ran_GTP_. Second, the compounds may interact with CRM1 outside of the NES-binding domain thereby inducing conformational changes precluding the assembly of a functional export complex. In order to achieve a cargo specific nuclear export inhibition, the compounds need to target putative cofactors required for the assembly of protein specific export complexes. Alternatively, and more likely the compounds may directly interact with the nuclear export signal sequence, thereby blocking the binding of the NES-containing cargo to the CRM1/Ran_GTP_ complex. Since NESs can be grouped into specific categories according to their activity *in vivo* (Figure 1c), these differences may represent an attractive opportunity to selectively interfere with export and the biological functions of proteins by the identification of such NES-specific inhibitors [26]. Currently, we are in the process of discriminating between these possibilities by investigating the inhibitory effects of the compounds on different shuttle proteins. Besides functional and biochemical assays, we are employing a set of translocation biosensors containing the NESes of proteins with high disease relevance in ArrayScan^®^-based primary screens (see [3,25,37].

Although “LMB-like” inhibitors that irreversibly bind and inactivate CRM1 will most likely not be used in therapeutic applications, they have been and will be used in the future as valuable chemogenomic tools to dissect the regulation and (patho)physiological relevance of nucleo-cytoplasmic transport. Moreover, compounds that are only transiently inactivating CRM1 and thus, may allow to interfere with nuclear export of regulatory proteins in a defined time window may have therapeutic potential, especially when combined with other targeted therapeutics, such as kinase inhibitors. Certainly, compounds that display a protein- or at least a protein class-specific inhibitory activity will most likely represent promising lead structures for the development of novel therapeutics for various diseases including cancer.

In summary, the kinetic biosensor assay presented allowed the high-throughput identification of novel inhibitors from the *ChemBioNet* compound collection, targeting the CRM1 nuclear export pathway. Our approach also represents a successful example for forward chemogenomics [27,28], which in combination with the recent technological and experimental developments in the area of reverse chemogenomics [41] will help to explore the intersection of the biological and chemical space. The detailed knowledge of the compounds' molecular mode of action will not only allow to identify chemical hit-to-lead scaffolds for future developments but will also aid in the building of a *Chem-Bio* data base to achieve an improved understanding of the chemical structure-biological function relationship.

## 

Supplementary Figure 1. (a) Prolonged incubation with LMB affects cell viability. 1×10^3^ A431_bio_ cells/well were seeded into 96-well plates and treated with the indicated concentrations of LMB or were mock treated (DMSO). ATP concentrations in cell lysates, reflecting cell viability, were determined after the indicated time points using the CellTiter-Glo^®^ Luminescent Cell Viability Assay (Promega, Madison, WI) as described [39]. Whereas LMB treatment for up to 5h did not show a significant effect, incubation for 24h resulted in a strong reduction in cell viability. No significant dose dependence was observed. RLU, relative light units; columns, mean; bars, SD. (b) The novel export inhibitors are active in cell culture models of different tumor types. Indicated cell lines transiently expressing the RevNES biosensor were treated with the indicated compounds (25 μM). The localization of the biosensor is visualized in living cells by fluorescence microscopy 5 h after mock (DMSO), 2 h after LMB treatment and at time points when the compounds displayed their maximum inhibitory activity (T_opt_). All export inhibitors blocked export in FaDu (head and neck cancer cell line), MDA-MB-231 (breast cancer cell line) or RKO (colon cancer cell line) cells. Scale bar, 10 μm.

## Figures and Tables

**Table 1.** Translocation screen summary and assay performance data.

Compound parameters		
	*N° compounds*	*% of total*

Screened	16,671	100%
Rescreened	120	0.72%
Validated	11	0.07%

Screening parameters		
*Parameter*	*Value*	

# plates	48	
Total scan time	56h	
Scan time/well	11s	
Fields/well	4	
Average valid objects/well	470	

Z' factor calculation		
*Formula*	*Z' factor*	

CIRC_GFP_	0.71	
Ring_GFP_	−0.22	
CIRC_GFP_/ Ring_GFP_	0.37	
CIRC_GFP_ - Ring_GFP_	0.77	
