# Supplementary figures and images for: Translocation Biosensors – Cellular System Integrators to Dissect CRM1-Dependent Nuclear Export by Chemicogenomics
Source: Sensors (Basel). 2009 Jul 9;9(7):5423–45. doi: 10.3390/s90705423 (PMC3274152; doi:10.3390/s90705423)

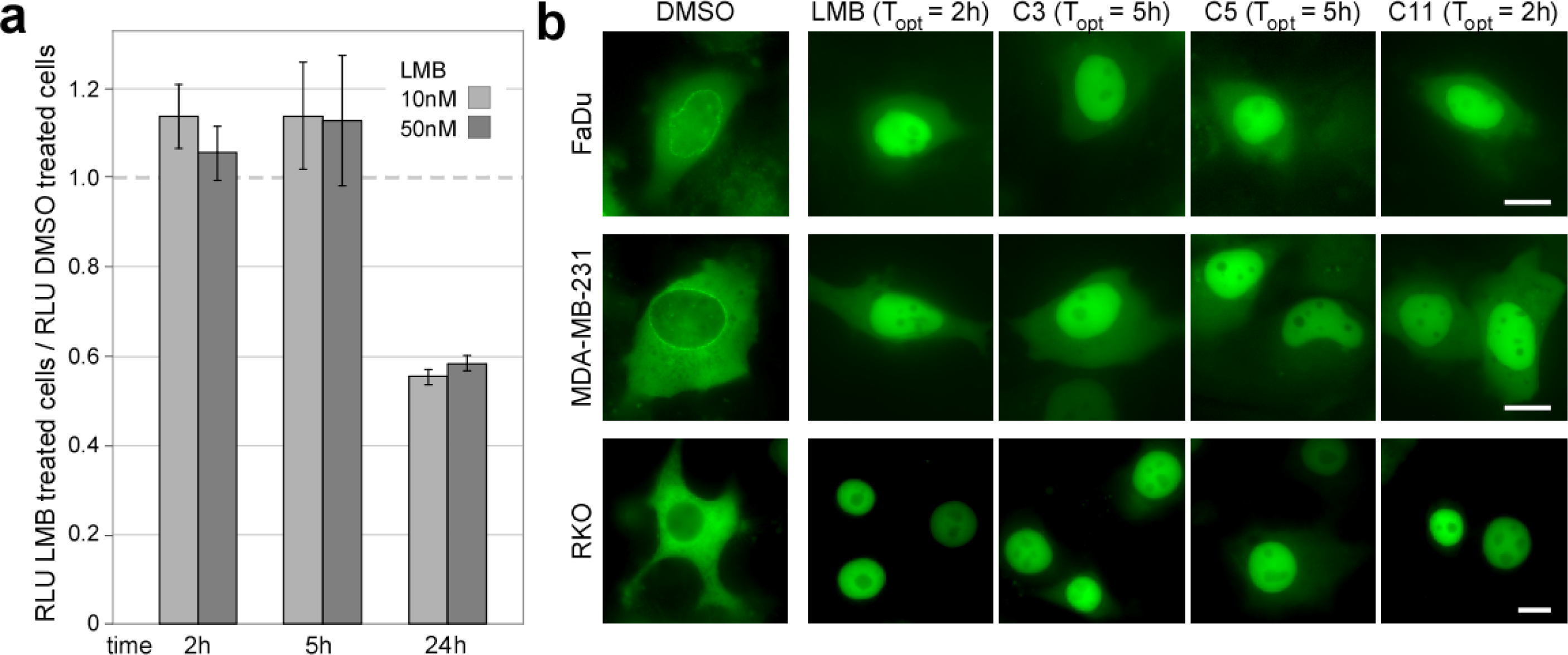

Supplement: Supplementary Figure 1. — (a) Prolonged incubation with LMB affects cell viability. 1×10^3^ A431bio cells/well were seeded into 96-well plates and treated with the indicated concentrations of LMB or were mock treated (DMSO). ATP concentrations in cell lysates, reflecting cell viability, were determined after the indicated time points using the CellTiter-Glo® Luminescent Cell Viability Assay (Promega, Madison, WI) as described [39]. Whereas LMB treatment for up to 5h did not show a significant effect, incubation for 24h resulted in a strong reduction in cell viability. No significant dose dependence was observed. RLU, relative light units; columns, mean; bars, SD. (b) The novel export inhibitors are active in cell culture models of different tumor types. Indicated cell lines transiently expressing the RevNES biosensor were treated with the indicated compounds (25 μM). The localization of the biosensor is visualized in living cells by fluorescence microscopy 5 h after mock (DMSO), 2 h after LMB treatment and at time points when the compounds displayed their maximum inhibitory activity (Topt). All export inhibitors blocked export in FaDu (head and neck cancer cell line), MDA-MB-231 (breast cancer cell line) or RKO (colon cancer cell line) cells. Scale bar, 10 μm. [file sensors-09-05423s001.tif]
